# A 72-hour high fat diet increases transcript levels of the neuropeptide galanin in the dorsal hippocampus of the rat

**DOI:** 10.1186/s12868-015-0188-9

**Published:** 2015-08-11

**Authors:** Ling Gan, Emily England, Jeong-Yeh Yang, Natalie Toulme, Suresh Ambati, Diane L Hartzell, Richard B Meagher, Clifton A Baile

**Affiliations:** Veterinary Medicine Department, Rongchang Campus, Southwest University, Rongchang, Chongqing, People’s Republic of China; Animal and Dairy Science, University of Georgia, Athens, GA USA; Neuroscience Division Biomedical and Health Sciences Institute, University of Georgia, Athens, GA USA; College of Veterinary Medicine, University of Georgia, Athens, GA USA; Department of Genetics, University of Georgia, Athens, GA USA

## Abstract

**Background:**

Recent evidence identifies the hippocampus, a brain structure commonly associated with learning and memory, as key to the regulation of food intake and the development and consequences of obesity. Intake of a high fat diet (HFD) results in altered consumptive behavior, hippocampal damage, and cognitive deficits. While many studies report the effects of HFD after chronic consumption and in the instance of obesity, few examine the events that occur following acute HFD consumption. In this study, male rats were fed either a control diet (10% fat by kcal) or HFD (45% fat by kcal) for 72 h. At the end of the 72-h period, serum and tissues were collected and weighed. Brains were rapidly frozen or formalin-fixed in preparation for qRT-PCR or immunohistochemistry, respectively.

**Results:**

Acute intake of HFD resulted in higher serum levels of leptin and cholesterol, with no significant changes in final body weight or adipose tissue mass. In the dorsal hippocampus, transcription of the neuroprotective peptide *galanin* was significantly upregulated along with a trend for an increase in *brain*-*derived neurotrophic factor* and *histone deacetylase 2* in the rats fed HFD. In the ventral hippocampus, there was a significant increase in *histone deacetylase 4* and a decrease in *galanin receptor 1* in this group. Results from immunohistochemistry validate strong presence of the galanin peptide in the CA1/CA2 region of the dorsal hippocampus.

**Conclusions:**

These results provide evidence for a distinct response in specific functional regions of the hippocampus following acute HFD intake.

## Background

Overweight and obesity are at epidemic levels in the United States and rates are rising in other countries that have adopted a more Western diet [1]. Consumption of diets rich in saturated fatty acids is commonplace in the United States and is pinpointed as a major factor in the development of obesity and resulting metabolic disease states [2]. Excess intake of saturated fat has also been implicated in the development of neurodegenerative diseases such as Alzheimer’s [3, 4].

It is well established that the hippocampus is a major brain region involved in memory, particularly episodic and spatial memory [5]. However, the hippocampus is a heterogeneous structure with multiple projections to other areas of the brain involved in emotional motivation and feeding behaviors [6, 7]. A thorough review by Lathe highlighted the role of the hippocampus in monitoring the physiological environment and modulating an appropriate response, a major component of which is sensing of the endocrine and metabolic state of the blood and cerebral spinal fluid [8]. In fact, the blood–brain barrier in the vicinity of the hippocampus is particularly vulnerable to the exterior environment. Two studies have shown a reduction in blood brain barrier integrity in the vicinity of the hippocampus following high fat diet (HFD) consumption [9, 10]. Dietary fat and cholesterol are able to cross the blood–brain barrier and promote protein infiltration, a process that may contribute to the development of Alzheimer’s [9]. Behavioral effects can be observed rapidly after this HFD-induced insult. Impairments in hippocampal-specific spatial memory are observed after only 3–5 days on a high-energy diet (higher in saturated fat and glucose), with no effects of the diet on memory tasks that were not dependent on the hippocampus [11, 12]. This would suggest that the hippocampus responds more rapidly to dietary insult than other areas of the brain.

While many studies show the impact of chronic consumption of HFD on the molecular physiology of brain, few examine the acute response [13–16]. We investigated the effects of HFD consumption in a 72-h time window, which for humans might be the amount of time a typically healthy individual spends eating high fat foods over a holiday or while on vacation. Three recent studies from the University of Washington showed that during the first 3 days of high fat feeding, hypothalamic inflammation, reactive gliosis and astrocytosis were present in both rats and mice at this time point, and that similar gliosis occurred in obese human subjects [12, 16, 17]. Other experiments involving 72-h HFD consumption in rodent models have shown increased body weight, increased adipose tissue mass, and increases in markers of inflammation and oxidative stress in the brain, liver, and adipose tissue [12, 18, 19]. Little is known about changes in hippocampal gene expression following acute HFD intake.

It is now widely accepted that changes in the physical environment may be rapidly recorded in different regions of the brain by epigenetic mechanisms involving changes in chromatin structure [20]. For example, acetylation of nucleosomal histones is sensitive to dietary change and correlates with altered gene expression in a very rapid timeframe [21, 22]. Consequently, we chose to examine the expression levels of several enzymes that alter chromatin structure, as well as trophic factors, hormone receptors, genes associated with obesity and inflammation, and the neuropeptide galanin. Galanin is a small, highly conserved neuropeptide that is expressed throughout the mammalian central and peripheral nervous system [23, 24]. It is known to be involved in feeding, the regulation of metabolism, neuronal excitability, neuroprotection, cognition, and stress, to name a few [25, 26]. In feeding studies, galanin is shown to be orexigenic, particularly stimulating the consumption of fat and intake of fat will alter levels of *galanin* mRNA in the hypothalamus [27]. Early changes in galanin expression in the hippocampus as well might help explain why animals do not self-regulate when offered a high fat diet, and will continue to consume until reaching a state of obesity.

We hypothesized that the gene expression profile of rats fed a high fat diet for 72 h would be indicative of hippocampal damage and a propensity towards obesity, meaning that we expected to see a downregulation of neurotrophic and neuroprotective factors, an upregulation of certain epigenetic enzymes, and a downregulation of insulin and leptin receptors in rats fed a high fat diet for 72 h. However, our results show a distinct response in specific functional poles of the hippocampus following acute HFD intake, with potential mediators of neuroprotection at play in the dorsal hippocampus.

## Results

### Energy intake, body and tissue weights

**Fig. 1:**
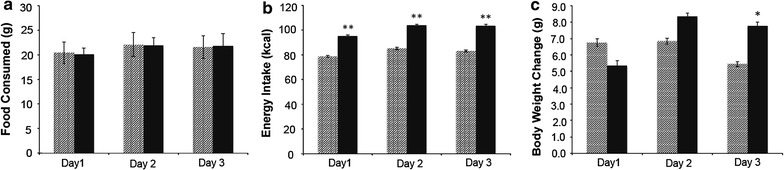
Energy Intake, body and tissue weights. Eight to ten week-old male Long-Evans rats were fed diets either low in fat (*light bars*, n = 10) or high in fat (*dark bars*, n = 10) for 72 h. Food intake in grams (**a**), kilocalories (**b**), as well as body weight (**c**) were measured daily following 24 h (1 day), 48 h (2 days), and 72 h (3 days) on the diet. Statistics were performed using two-way ANOVA with post hoc Tukey’s HSD and Levene’s test for equality of variances. A p value of p < 0.05 is denoted by *asterisk* and a p value of p < 0.01 is denoted by *double asterisk*.

Male rats given a HFD ad libitum (45% kcal from fat) for 72 h did not consume more food in grams than their control-fed counterparts (Fig. 1a), but they also did not reduce their food intake to account for increased caloric value of the HFD and consumed more energy on each day of the study (p < 0.001; Fig. 1b). Results from a two-way ANOVA show that both dependent variables, food intake and energy intake, were normally distributed and that there was homogeneity of variance between groups as assessed by Levene’s test for quality of variances. There was not a significant interaction between the effects of day and treatment on food intake (g) [F(2,48) = 0.213, P = 0.809], or energy intake (kcal) [F(2,48) = 0.197, P = 0.822]. HFD-fed rats did not weigh significantly more than rats fed a control diet (10% kcal from fat) at the conclusion of the study (Table 1), although they did gain more weight during the last 24-hour period (day 3) of the study (p < 0.05, Fig. 1c). There was a significant interaction between the effect of day and treatment on the weight gain (g) of the rats [F(2,48) = 0.197, P = 0.020]. There was no significant difference in the raw weights of white or brown adipose depots either alone or when combined (Table 1), or when they were calculated as a proportion of body weight (not shown). Liver weight was reduced in HFD-fed rats (Table 1) and this change was significant as a proportion of body weight (p < 0.05, not shown).

**Table 1:** Mean body parameters

Measurement	Control diet	High fat diet	p value
Body weights			
Body weight, initial (g)	299.4 ± 0.46	298.7 ± 0.66	0.802
Body weight, final (g)	318.4 ± 0.445	320.1 ± 0.772	0.528
72-Hour weight gain (g)	19.05 ± 0.36	21.28 ± 0.30	0.093
Final tissue weights			
Inguinal adipose tissue (g)	6.32 ± 0.070	6.71 ± 0.101	0.326
Epididymal adipose tissue (g)	3.61 ± 0.049	3.89 ± 0.072	0.335
Retroperitoneal adipose tissue (g)	3.31 ± 0.029	3.53 ± 0.081	0.444
Omental adipose tissue (g)	0.183 ± 0.011	0.155 ± 0.004	0.460
Pericardial adipose tissue (g)	0.586 ± 0.009	0.534 ± 0.015	0.380
Total white adipose tissue (g)	14.2 ± 0.152	14.8 ± 0.178	0.376
Subscapular brown adipose tissue (g)	0.43 ± 0.006	0.43 ± 0.008	0.994
Liver weight (g)	14.4 ± 0.131	13.3 ± 0.080	0.050

After 72 h of control (n = 10) or high fat diet (n = 10), animals were weighed and fasted for 2 h before sacrifice. Inguinal, epididymal, retroperitoneal, omental, and pericardial white adipose depots; subscapular brown adipose; and livers were dissected and weighed. Statistics were performed using t test.

### Blood and serum measures

After 72-h of a HFD, serum leptin (p < 0.01) and total serum cholesterol (p < 0.05) levels were significantly increased relative to controls (Table 2). There was no significant difference in either blood glucose or serum insulin levels between control- and HFD-fed rats (Table 2).

**Table 2:** Blood and serum measures at endpoint

Measurement	Control diet	High fat diet	p value
Blood glucose (mg/dl)	123.0 ± 1.12	122.7 ± 1.15	0.952
Serum insulin (ng/ml)	2.40 ± 0.07	2.16 ± 0.05	0.365
Serum leptin (ng/ml)	1.55 ± 0.00	1.57 ± 0.00	0.005**
Serum cholesterol (mg/dl)	112.4 ± 0.96	128.0 ± 1.78	0.026*

After 72 h of control (n = 10) or high fat diet (n = 10), animals were fasted for 2 h before sacrifice. At the time of sacrifice, trunk blood was used to measure blood glucose. Blood was allowed to clot for collection of serum. Serum insulin and leptin were measured via ELISA and total serum cholesterol was measured chemically. Statistics were performed using t test. A p value of p < 0.05 is denoted by * and a p value of p < 0.01 is denoted by **.

### Gene expression

**Fig. 2:**
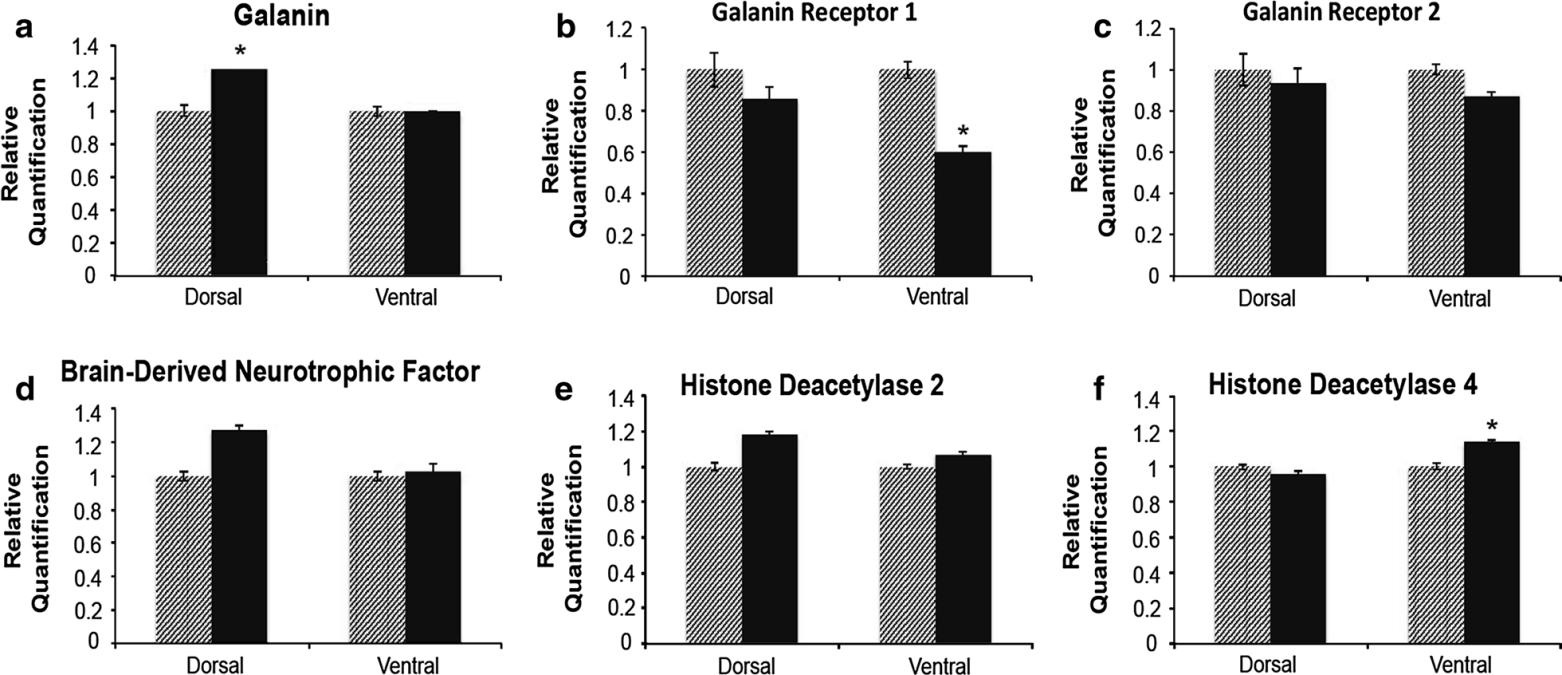
Gene expression. Dorsal and ventral hippocampal qRT-PCR results for *galanin* (**a**), *galanin receptor 1* (**b**), *galanin receptor 2* (**c**), *brain*-*derived neurotrophic factor* (**d**), *histone deacetylase 2* (**e**), and *histone deacetylase 4* (**f**). GAPDH was used as an endogenous control. *Light bars* indicate control diet (n = 10) and *dark bars* indicate high fat diet (n = 10). Statistics were performed using t test. A p value of p < 0.05 is denoted by *asterisk* and a p value of p < 0.01 is denoted by *double asterisk*.

In the dorsal hippocampus, there was a significant 20% increase in transcript levels of the neuropeptide *galanin* (p < 0.0488; Fig. 2a). There was no change in transcript levels of either *galanin receptor 1* or *2* (p = 0.590 and p = 0.818 respectively; Fig. 2b, c). Also in the dorsal hippocampus there was a trend for an increase in *brain*-*derived neurotrophic factor* and *histone deacetylase 2* (p = 0.0593 and p = 0.0604 respectively; Fig. 2d, e) in HFD-fed rats compared to controls.

In the ventral hippocampus of HFD-fed rats, there was a significant 66% decrease in transcript levels of *galanin receptor 1* (p = 0.0153; Fig. 2b) and a significant 14% increase in transcript levels of *histone deacetylase 4* (p = 0.0497; Fig. 2f). There was no significant difference in levels of *galanin* or *galanin receptor 2* transcripts compared to controls (p = 0.978 and p = 0.220 respectively, Fig. 2a, c), or in transcript levels of other genes tested (Table 3) in either the dorsal or ventral hippocampus.

**Table 3:** Gene primers

Gene symbol	Gene name	Forward and reverse
*GAPDH*	*Glyceraldehyde 3*-*phosphate dehydrogenase*	GGGAAACCCATCACCATCTT
		CCAGTAGACTCCACGACATACT
*BDNF*	*Brain*-*derived neurotrophic factor*	GAGACAAGAACACAGGAGGAAA
		CCCAAGAGGTAAAGTGTAGAAGG
*FTO*	*Fat mass and obesity*-*associated protein*	CTGTGGAAGAAGATGGAGAGTG
		CAGGACGGCAGACAGAATTT
*GAL*	*Galanin*	CCATTGACAACCACAGATCATTTA
		CAACACTTCCTAGTCTCCCTTC
*GALR1*	*Galanin receptor 1*	GTTCCCATAGGTGTACAGAGTTC
		GGTGTCTTAGTCCACAGGATTAC
*GALR2*	*Galanin receptor 2*	GGACCAAAGGGCATCTAACA
		CCTACAATCCTCGGTCTTTAGC
*HAT1*	*Histone acetyltransferase 1*	TGTTTCTCCCGGGAAAGATTAC
		CCCGTCTAGCATGTTGCTTAT
*HDAC2*	*Histone deacetylase 2*	CTGTCAAAGGTCACGCTAAATG
		GTCCAACATCGAGCAACATTC
*HDAC4*	*Histone deacetylase 4*	AGCTGCAGGAGTTTGTTCTC
		CTGTGCTGTGTCTTCCCATAC
*INSR*	*Insulin receptor*	CCCTGTGACCCATGAAATCTT
		CGCCGATAGCTCACTTCATATAG
*OBRB*	*Leptin receptor, long form*	GGTTGGATGGACTAGGGTATTG
		CAGAATTCAGGCCCTCTCATAG
*ORXR*	*Orexin receptor*	CTCCTCATCGTGACACTGAAAG
		GAGGAAGAGAAACTCCCACAAG
*RHEB1*	*Ras homolog enriched in brain 1*	GAGCCCACCACCTCAATAAT
		GGGAAAGTGCAGATACCGATTA
*SOCS3*	*Suppressor of cytokine signaling 3*	ACCTTTCTTATCCGCGACAG
		CACTGGATGCGTAGGTTCTT
*SYN1*	*Synapsin 1*	GGACGGAAGGGATCACATTATT
		ACCACAAGTTCCACGATGAG

Sequences for all primers used in qRT-PCR reactions.

### Galanin immunohistochemistry

**Fig. 3:**
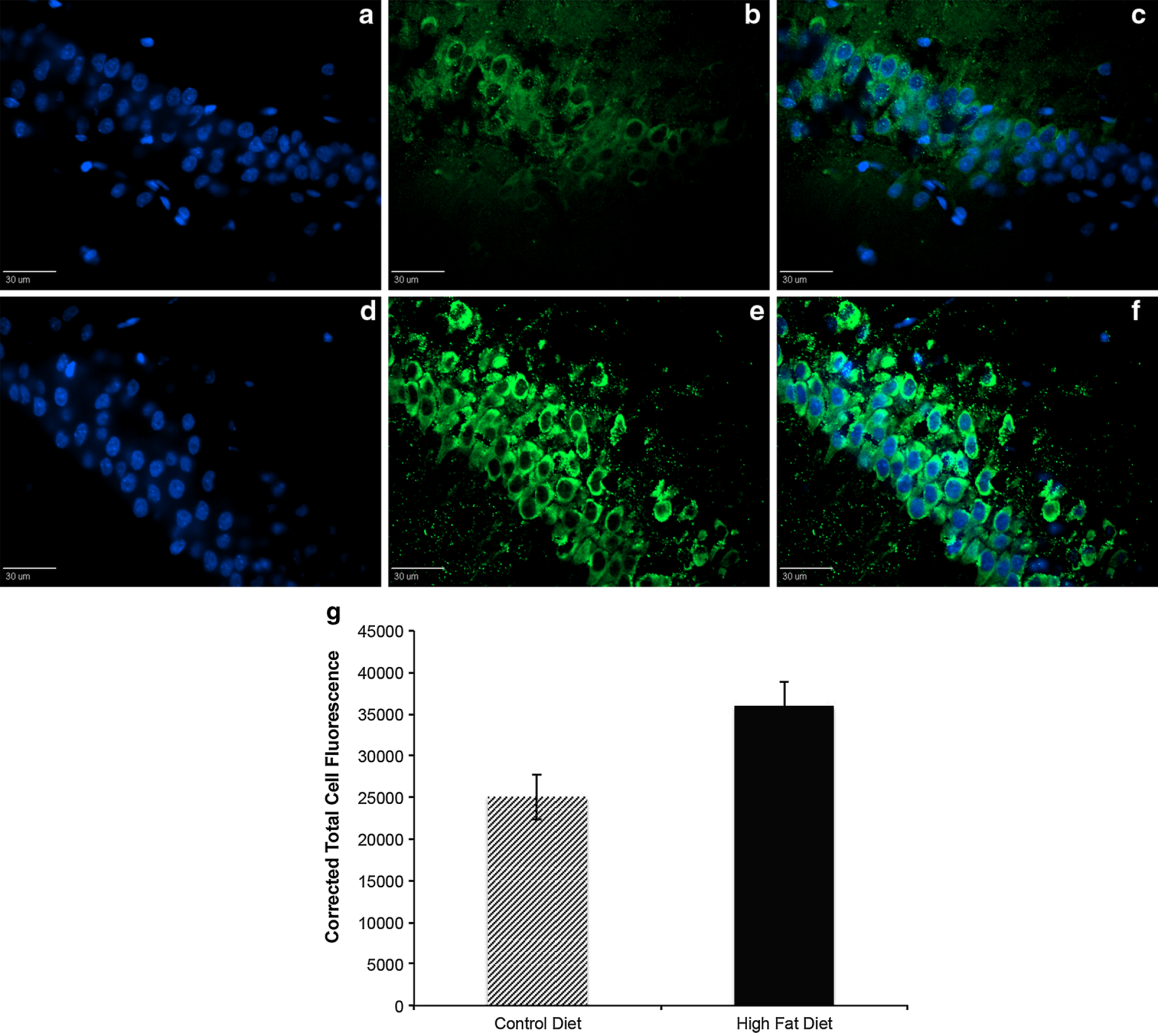
Galanin immunohistochemistry. Galanin immunostaining of dorsal hippocampal CA1/CA2 regions in one control (**a**–**c**) and one high fat-fed (**d**–**f**) rat after 72 h. *Scale bars* set to 30 μm. **a**, **d** show DAPI staining in *blue*, **b**, **e** show galanin staining in *green*, and in **c**, **f** the images are merged. Quantification of GFP signal for high fat fed (n = 3) and control fed (n = 4) rats using Image J software (**g**). *Light bars* indicate control diet and *dark bars* indicate high fat diet. Statistics were performed using t test.

Immunostaining for the galanin protein in brain sections including the dorsal hippocampus showed a strong perinuclear pattern of staining in the pyramidal cells of the CA1 and CA2 regions (Fig. 3a–f), though differences in fluorescence intensity levels between treatment groups did not reach statistical significance (p = 0.22, Fig. 3g).

## Discussion

High intake of saturated fatty acids is identified as a factor leading to cognitive impairment later in life [28]. In this study, we found that rats fed a HFD for only 72 h had significantly higher serum cholesterol than control-fed rats. Increased permeability of the blood–brain barrier has also been observed in rabbits with increased circulating cholesterol [29, 30]. Other studies have linked high fat and cholesterol diets to damage of the blood–brain barrier with corresponding impairments in hippocampal-dependent memory tasks [10].

Here we provide evidence that acute consumption of a HFD impacts the hippocampus. From our qRT-PCR results, we found that levels of *histone deacetylase* (*HDAC*) transcript were trending upward in the dorsal hippocampus and significantly increased in the ventral hippocampus of HFD-fed rats. The epigenetic machinery tasked with modulating acetylation and methylation levels of DNA and histones, thus affecting gene expression, can be rapidly influenced by environment. HDAC activity is increased in instances of neuronal cell death and administration of HDAC-inhibitors has been proposed in the treatment of Alzheimer’s disease [31, 32]. A recent review by Mielcarek et al. [33] highlights the role of HDAC4 in neuronal cell death. Interestingly, the activity of HDAC4 is regulated by its translocation from the cytoplasm to the nucleus. Wang et al. showed that in *Drosophila*, an important model organism for the study of obesity and diabetes, feeding activated the AMPK-family kinase SIK3, which phosphorylated HDAC4 and promoted its sequestration in the cytoplasm [34]. While upregulation of HDAC4 mRNA in the ventral hippocampus could be evidence of a compensatory response related lack of active HDAC4 in the nucleus of fed rats, the feeding-induced signal for this pathway was reported to be insulin [34], which was not significantly increased in our HFD-fed animals. Additionally, rats in our study were fasted for 2 h before tissues were collected. Knowing that the mediator of apoptosis capsase-3 [17] and the inflammatory stimulator lipopolysaccharide (LPS) [35] are both upregulated by HFD, and that both of these signals trigger the degradation of HDAC4 [33], it is also possible that HFD-induced degradation of HDAC4 protein triggered the upregulation of HDAC4 transcripts that we observed in the ventral hippocampus of our HFD-fed rats.

Importantly we show that transcript levels of *galanin* are upregulated in the dorsal hippocampus after just 72 h on a HFD. One of the many roles galanin is known to play in the central nervous system is that of a neurotrophic and neuroprotective factor [27, 36–39]. Specifically in the hippocampus, galanin has been implicated as having both beneficial [26, 40, 41] and detrimental [25, 41, 42] effects regarding learning and memory, due mainly to its ability to regulate neural activity in the hippocampus through modulation of cholinergic transmission [43]. *Galanin* is also rapidly upregulated in septohippocampal neurons following either lesion of the area or blockade of neuronal activity [44]. Additionally, as previously stated, galanin is particularly involved in promoting the intake of fat [27], however we would expect this function of galanin to be associated primarily with the ventral hippocampus and we did not see a significant increase in transcript levels in this region. Also, we saw an increase in *galanin* only in the dorsal hippocampus, not in the ventral hippocampus where there was the most significant increase in *HDAC* transcripts. While the ventral hippocampus is reportedly involved in emotion and motivation, the dorsal hippocampus and its connections to the frontal cortex are critical for learning and memory [6, 7], and it is important to note that the function of galanin depends largely on the region of the brain in which it is acting [45–48]. Though our immunohistochemical results do not show a statistically significant increase in galanin protein signal in the dorsal hippocampus, there are some potential explanations for this discrepancy that would not necessarily negate our conclusions about *galanin* mRNA: (1) We are examining only an acute response (3 days) and the transcription of mRNA is more rapid than the translation of protein, (2) Immunohistochemistry is not as reliable a method for quantifying protein as other methods such as Western blot, therefore these results are most useful to display the location of galanin in the dorsal hippocampus, and (3) The small sample size (n = 5) of our immunohistochemistry experiment was likely not powerful enough to generate significant results. Regional- and cell type-specific studies, along with more extensive protein analysis, will be needed to further elucidate galanin’s actions in the brain in response to acute HFD.

In addition to the significant upregulation of *galanin* in the dorsal hippocampus, this study also demonstrated a trend for an increase in the transcript levels of *brain*-*derived neurotrophic factor* (*BDNF*) in the same region. BDNF is known to enhance hippocampal function by increasing neurogenesis and neurite growth, enhancing long-term potentiation and spatial memory, protecting the hippocampus against excitotoxic injury, and for its involvement in neurodevelopment [26, 49, 50]. This finding is surprising considering the majority of the literature regarding BDNF and HFD points to a decrease in *BDNF* transcription following HFD, however in these experiments the animals consumed the HFD for 5 weeks or longer [50–52]. The upregulation of *BDNF* and *galanin* in the dorsal hippocampus suggests that the acute response to HFD is entirely different than the long-term response and requires further study.

Despite the increase in *galanin* transcript in the dorsal hippocampus, our qRT-PCR results did not show changes in either *galanin receptor 1* (*GALR1*) or *galanin receptor 2* (*GALR2*) transcripts in this region. We did observe a decrease in the transcript levels of *GALR1* in the ventral hippocampus. All three *galanin receptor* genes (1, 2, and 3) are expressed in both dorsal and ventral hippocampus [53, 54], with *GALR1* having exceptionally high expression in the ventral hippocampus when compared to the dorsal hippocampus and the brain as a whole [46, 47]. Pharmacological studies point to GALR1 as playing a larger role in feeding than either GALR2 or GALR3 [54]. GALR3 has a lower affinity for galanin than GALR1 or GALR2 and is postulated to have a greater role in the periphery; therefore it was not chosen for analysis in this study [54, 55]. Evidence that galanin serves different functions in the dorsal versus ventral hippocampal regions is supported by our findings and leads us to believe that HFD differentially regulates the galanin pathway in these two regions [46, 48]. Galanin administration has completely opposite effects depending on the site of infusion, for example, decreasing basal acetylcholine release in the dorsal hippocampus and increasing release in the ventral hippocampus [48]. With GALR1 being reduced in the ventral hippocampus, we might expect that release of galanin here would be increased, causing a compensatory downregulation of the receptor. However, since we did not see a significant increase in galanin mRNA in the ventral hippocampus, meaning that this increased galanin is not coming from the ventral hippocampus itself, we hypothesize that there could be an increase in galanin peptide release into the ventral hippocampus coming from other areas of the brain, such as the locus coeruleus. The locus coeruleus is one of the major galanin-producing nuclei in the brain and sends direct galanergic projections to both the dorsal and ventral the hippocampus. However, we cannot verify this hypothesis with the remaining brain tissue from our study and do not know of studies showing a direct connection between high fat diet and increased galanin release from these neurons. Additionally, dopamine, the neurotransmitter most associated with motivation and reward, is activated following consumption of HFD and has long provided evidence for the rewarding effects of high fat and highly palatable foods [56, 57]. A recent study by Valdivia et al. showed that dopaminergic neurons in the ventral tegmental area (VTA), a reward-related brain area, are activated following only 2 h of HFD intake [58]. Dopamine receptor stimulation has the ability to modulate the effects of GALR1 activation and, interestingly, dopamine-galanin heteromers in the hippocampus are found only in the ventral pole, not the dorsal [46]. It is possible that an increase in VTA dopamine release following HFD could project, via the mesolimbic dopamine pathway, to the ventral hippocampus and result in a feedback downregulation of GALR1-expressing dopamine-responsive neurons in this area.

A possible confound in our interpretation of these results lies in the issue of novelty. In this experiment, animals were maintained on the control diet prior to start of the experiment and only the high fat group was switched to a new diet. To our current knowledge, no connections have been made between galanin and exposure to a novel food. However, the hippocampus itself responds strongly to a variety of novel events [59]. Learning and memory are critical components of feeding: an encounter with a food item prompts an animal to determine if he’s ever encountered it before, and to remember the experience with the food item in case he is to encounter it again in the future [60]. Due to its classical role in learning and memory, the hippocampus cannot be separated from these processes. Importantly though, the issue of novelty also cannot be separated from the human experience with exposure to a highly palatable food. In cases of short-term high fat feeding in humans, such as in instances of vacation or holidays, exposure to and consumption of novel foods is likely a major contributor to caloric intake, and likely causes activation of similar brain pathways as does our model.

In this study we showed that rats fed a HFD for 72 h had significantly higher serum leptin than rats fed a control diet. Importantly, this change was independent of a significant increase in fat mass. Many studies highlight a neuroprotective role for leptin in the central nervous system. Leptin reduces neuronal apoptosis, increases cell survival and proliferation, and reduces damage caused by stroke; specifically in the hippocampus, leptin facilitates plasticity [61, 62]. A few studies have shown evidence of leptin receptors on both galanin- and BDNF-expressing neurons in the brain, particularly the hypothalamus, and that leptin indirectly mediates both galanin and BDNF release [63–65]. It is possible that in this 72-hour HFD model, the upregulation of *galanin* and *BDNF* are being mediated through the observed increased circulating leptin, but further studies are needed to determine the presence of this molecular interaction in hippocampal neurons. Chronic HFD consumption results in central and peripheral leptin resistance, thereby preventing the neuroprotective role of leptin in obesity and potentially contributing to the downregulation of *BDNF* after chronic HFD consumption, as mentioned earlier.

Our observance of a reduction in liver weight after 72 h of high fat diet is surprising, however previous studies by Miller et al. and Ren et al. shed light on a possible mechanism. In those experiments, 72 h of high fat feeding in rodents down-regulated hepatic lipogenesis, possibly due to an inhibitory effect of the dietary fat on certain hepatic enzymes [19, 66]. Additionally, compared to the HFD group, the control animals consumed a greater proportion of their calories as carbohydrate, which is likely to have caused increased glycogen storage in control livers [67] compared to livers of HFD animals, another possible explanation for the differences in final liver weight between the groups. It is well known that chronic high fat diet promotes lipid accumulation in the liver, leading eventually to hepatic steatosis and non-alcoholic fatty liver disease, but our current findings along with the previous studies shed light on the important differences between acute and chronic exposure models.

## Conclusion

In conclusion, this study demonstrates a unique acute response to HFD consumption in the hippocampus of the rat. Prior to significant increases in fat mass or body weight, gene expression in the hippocampus is altered in a way that reflects a distinct response in specific functional poles of the hippocampus following acute HFD intake, with potential mediators of neuroprotection at play in the dorsal hippocampus. Future studies should examine structural changes in the hippocampus at this time point to determine the level of HFD-induced insult after 72 h as well as examine the response of females to acute HFD, as there is evidence for sex differences in this model [19].

## Methods

### Animals and feeding

Twenty male, 8–10 week-old Long-Evans rats (200–250 g) were purchased from Harlan (Indianapolis, IN, USA). Upon arrival rats were housed individually and adapted to the rodent facility and to a low-fat control diet (Control, Table 4, D12450B; Research Diets; New Brunswick, NJ, USA) for 11–16 days. Rats were weight-matched and either maintained on the control diet (n = 10) or switched to a high-fat diet (HFD; n = 10; Table 4, D12451; Research Diets; New Brunswick, NJ, USA). Rats had access to the diets and water ad libitum throughout the experiment. Food intake and body weight were monitored daily. Rooms were temperature (22 ± 2°C) and humidity controlled and kept on a 12:12-h light/dark cycle. All institutional and national guidelines for the care and use of laboratory animals were followed. All protocols for this experiment were approved by the University of Georgia Institutional Animal Care and Use Committee (AUP #A2013 09-005-Y1-A0) prior to the start of this experiment.

**Table 4:** Diet composition (research diets)

Diet	Control	High fat diet
Catalog number	D12450B	D12451
Form	Pelleted	Pelleted
Macronutrients (kcal%)		
Total fat	10	45
Soybean oil	5.5	5.5
Lard	4.4	39.4
Protein	20	20
Carbohydrate	70	35
Cholesterol	167.8 mg/kcal	54.4 mg/kcal
Total kcal/gm	3.85	4.73
Fat and carbohydrate content (kcal%)		
Soybean Oil	5.55	5.55
Lard	4.44	39.3
Corn Starch	31.1	7.17
Maltodextrin 10	3.45	9.86
Sucrose	34.5	17.0

Description of diets provided to rats for the duration of the study (n = 10/diet).

### Tissue collection

After 72 h of dietary treatment, rats were fasted for 2 h then anesthetized with inhaled isoflurane anesthesia (2.5%) and euthanized by decapitation. Trunk blood was collected immediately for measurement of glucose (FreeStyle^**®**^ Lite Blood Glucose Monitoring System; Abbot Diabetes Care, Abbot Park, IL, USA) and then allowed to clot for 30 min before serum was collected for further analysis. The brain was removed from the skull and weighed. The left hemisphere was rapidly frozen on dry ice for RNA isolation and the right hemisphere was fixed in 4% formaldehyde (Avantor; Center Valley, PA, USA) for 28 h and flash frozen for immunohistochemistry. Inguinal, epididymal, retroperitoneal, omental, pericardial, and subscapular brown fat depots, along with the liver, were removed, weighed, and frozen in liquid nitrogen for long-term storage.

### ELISA

Serum insulin was measured using a rat/mouse-specific ELISA kit (EZRMI-13 K; Millipore; Billerica, MA, USA). Serum leptin was measured using a rat-specific ELISA kit (EZRL-83K; Millipore; Billerica, MA, USA). Total serum cholesterol was determined using a cholesterol reagent set (C7510; Pointe Scientific; Ann Arbor, MI, USA). All kits were used according to the manufacturers instructions and spectrophotometric measurements were made on a Flex Station 3 (Molecular Devices; Sunnyvale, CA, USA).

### Quantitative reverse transcription polymerase chain reaction

Total RNA from the dorsal and ventral hippocampus was isolated using E.Z.N.A. Microelute Total RNA Kit (Omega Bio-Tek; Norcross, GA, USA) and quantified using a Nanodrop spectrophotometer (ND-1000; Thermo Scientific; Wilmington, DE, USA). 100 ng of RNA went into each reverse transcription reaction using the High Capacity cDNA Reverse Transcription Kit (436814; Life Technologies; Grand Island, NY, USA) and a Thermocycler (Professional Thermocycler, Biometra; Goettingen, Germany) to synthesize cDNA. Using cDNA produced from a 5 ng equivalent per sample, expression levels of transcripts for *brain*-*derived neurotrophic factor (BDNF), fat mass and obesity*-*associated protein (FTO), galanin (GAL), galanin receptor 1 (GALR1), galanin receptor 2 (GALR2), histone acetyltransferase 1 (HAT1), histone deacetylase 2 (HDAC2), histone deacetylase 4 (HDAC4), insulin receptor (INSR), the long form of the leptin receptor (OBRB), orexin receptor (ORXR), ras homolog enriched in brain 1 (RHEB1), suppressor of cytokine signaling 3 (SOCS3), and synapsin 1 (SYN1)* were determined by qRT-PCR. Primers were designed using the NCBI online database (http://www.ncbi.nlm.nih.gov) and sequence specificity of each primer pair (Table 3) was confirmed using Primer-BLAST (http://www.ncbi.nlm.nih.gov/tools/primer-blast/index). Efficiency of primers for a single target sequence was determined by examining dissociation curves for each primer set and choosing the set that best amplified only our region of interest. In each RNA sample the level of *glyceraldehyde 3*-*phosphate dehydrogenase* (*GAPDH*) transcripts was used as an endogenous control. Quantitative real-time PCR was performed with SYBR green reaction mix (4309155; Invitrogen; Carlsbad, CA, USA) using a 7500 system from Applied Biosystems to determine cycle threshold (C_T_) values. For analysis of C_T_ values, each sample was run in triplicate and those triplicates were averaged to assign C_T_ values for each sample and each gene. Relative quantity was determined using the ddCt method [68].

### Immunohistochemistry

Ten μm-thick coronal brain sections taken on a cryostat (CM3050; Leica; Buffalo Grove, IL, USA) were used for immunofluorescence analysis to examine galanin (1:200 dilution, T-4334; Peninsula Laboratories T-4334; San Carlos, CA, USA) immunoreactivity in the hippocampus. Tissues were washed with PBST (PBS + 0.1% Triton X100) prior to antigen retrieval with 10 mM sodium citrate (pH 6.0). Tissues were blocked in 3% PBST (PBS + 3% BSA + 0.4% Triton X100) for one hour before being incubated with the primary antibody overnight in a humidified chamber at 4°C. The next day tissues were washed with PBST then incubated with the secondary antibody (1:500, Alexa 488; Abcam; Cambridge, MA, USA) and DAPI (1 mg/ml diluted 1:500; Thermo Scientific; Waltham, MA, USA). Slides were washed again and coverslips were mounted with glycerol (G7893, 70% in water; Sigma-Aldrich; St. Louis, MO, USA). Images were captured on an Olympus IX81 Motorized Inverted Fluorescent Microscope (Center Valley, PA, USA) and quantified by calculating corrected total cell fluorescence [CTCF = Integrated density − (area of selected cell × mean fluorescence of background readings)] using Image J software (NIH; Bethesda, MD, USA).

### Statistical analysis

The data are presented as the mean ± standard error of the mean (SEM) for all measurements. A *t* test (independent, by groups) was used to compare values between the Control and the HFD groups using Statistica software 7.1 (StatSoft; Tulsa, OK, USA). For food intake, energy intake, and weight gain over 3 days, a two-way ANOVA was used to examine the effects of day and treatment, with variance between groups assessed by Levene’s test for quality of variances and post hoc Tukey’s HSD test using SPSS Statistics 20 (IBM; New York City, NY, USA). A value of p < 0.05 is denoted with * while a value of p < 0.01 is denoted with **.

## Endnote

Please note that Dr. Clifton A. Baile died on May 19, 2014, close to the completion of this work.
